# Slow Spin Dynamics in Superconducting Ca_0.9_Ce_0.1_Fe_2_As_2_

**DOI:** 10.1038/srep10700

**Published:** 2015-05-29

**Authors:** K. Nadeem, W. Zhang, D. Y. Chen, Z. A. Ren, X. G. Qiu

**Affiliations:** 1 Beijing National Laboratory for Condensed Matter Physics, Institute of Physics, Chinese Academy of Sciences, P.O. Box 603, Beijing 100190, China; 2 Department of Physics, International Islamic University, H-10, Islamabad 44000, Pakistan; 3 School of Physical Science and Technology & Collaborative Innovation Center of Suzhou Nano Science and Technology, Soochow University, Suzhou 215006, China

## Abstract

Slow spin dynamics has been observed in superconducting under-doped Ca_0.9_Ce_0.1_Fe_2_As_2_ single crystal. Below 100 K, the system exhibits hysteresis in the cooling and warming protocols of temperature dependent resistivity due to first order tetragonal to orthorhombic structural transition with simultaneous magnetic transition from paramagnetic to spin density wave antiferromagnetic state of the iron (Fe) ions. Zero field cooled/field cooled (ZFC/FC) magnetization curves showed splitting at 32 K followed by a sharp increase of the FC curve and then FC plateau at low temperatures. Slow spin relaxation in both the ZFC and FC protocols was observed which is typical for spin-glass system. The system also showed features analogue to spin-glass behavior such as ZFC peak, FC plateau, ZFC slow spin relaxation, magnetic hysteresis, and ZFC ac memory effect. The spin-glass like behavior was rather weak and vanished at higher fields. The origin of the slow spin dynamics could be the inhomogeneous distribution of the cerium (Ce) spins ordered along the c-axis OR interactions between Fe and Ce spins which lead to magnetic frustration of Ce spins. All these findings support the coexistence of slow spin dynamics of Ce spins and superconductivity in Ca_0.9_Ce_0.1_Fe_2_As_2_ single crystal.

High T_c_ superconductivity gained exponential growing interest among the scientific community after the discovery of iron-based superconductor (FeSC)^1^. Since then different species of FeSC have been discovered^2^,^3^,^4^,^5^. The superconductivity in FeSC could be achieved by either appropriate hole/electron doping OR applying pressure^4^,^6^,^7^. The FeSCs exhibit layered structure similar to cuprates and are poor metals with spin density wave (SDW) antiferromagnetic (AFM) order of the Fe spins in the ab-plane. The AFM order gets suppressed upon appropriate doping or applying pressure and superconductivity emerges at the expense of antiferromagnetism. The most striking feature of FeSCs is the coexistence of magnetism and superconductivity^8^,^9^, which also differentiates them from cuprates^10^,^11^. Analysis of the magnetic ordering and its coexistence with superconductivity can contribute to the understanding of the pairing mechanism in high T_c_ superconductors^12^. Different types of magnetic orders such as ferromagnetism, antiferromagnetism and spin-glass are reported in different types of FeSCs. Luo *et al.*^8^ reported short range incommensurate antiferromagnetic order in superconducting BaFe_2-x_Ni_x_As_2_. Ren *et al.*^13^ reported the coexistence of ferromagnetism and superconductivity in EuFe_2_(As_0.7_P_0.3_)_2_ superconductor. Luo *et al.*^14^ reported the interplay between superconductivity and cerium (Ce) ions magnetic order in CeFeAs_1−*x*_P_*x*_O_0.95_F_0.05_ superconductor. Drew *et al.*^15^ reported the coexistence of disordered magnetism and superconductivity in SmFeAsO_1-*x*_F_*x*_ superconductor. Laplace *et al.*^16^ reported the incommensurate magnetic order in Ba(Fe_1−x_Co_x_)_2_As_2_ superconductor which competes with the superconducting state. Ma *et al.*^17^ reported the coexistence of superconductivity and antiferromagnetism in Ba(Fe_1-x_Ru_x_)_2_As_2_ superconductor. Spin-glass behavior due to spin disorder and magnetic frustration below a certain freezing temperature^18^,^19^ has been also reported in few FeSCs e.g., P doped EuFe_2_As_2_^20^, Co doped BaFe_2_As_2_^21^, and Se doped FeTe^22^.

The MFe_2_As_2_ (M122) type superconductors with M = Ba, Ca, Eu, and Sr consist of alternating FeAs and M layers^23^. The coupling between FeAs layers has been reported in rare-earth doped CaFe_2_As_2_ superconductors^24^. The inter- or/and intra-layer coupling can be controlled by using different types of dopants or applied pressure^25^. The disorder and magnetic frustration can be present within the inter- or intra-layers. Around 170 K, CaFe_2_As_2_ parent compound simultaneously exhibits tetragonal (T) to an orthorhombic and paramagnetic to antiferromagnetic ordered phase transitions. However another structural phase transition from tetragonal (T) to collapse tetragonal (cT) phase is also reported in CaFe_2_As_2_ by applying suitable pressure OR chemical doping^26^,^27^,^28^. The doping of rare-earth (La, Ce, Pr, and Nd) ions in CaFe_2_As_2_ can be used to tune the cT phase, magnetic phase and superconducting state^24^. Therefore the rare-earth doped CaFe_2_As_2_ superconductors are interesting candidates to investigate the coexistence of slow spin dynamics and superconductivity. The most interesting region for the coexistence of superconductivity and magnetism in iron pnictides is the under-doped region where both magnetism and superconductivity can coexist. In the over-doped region, there is a possibility of formation of magnetic clusters which can give a collective magnetic response^29^. In this article, we have investigated the coexistence of slow spin dynamics and superconductivity in under-doped Ca_0.9_Ce_0.1_Fe_2_As_2_ single crystal.

## Experimental

The Ca_0.9_Ce_0.1_Fe_2_As_2_ single crystals were grown by using FeAs self-reflux method as adopted in Ref. 24. Structural phase was identified by X-ray diffraction (Rigaku D/MAX-Ultima III) by using Cu Kα (λ = 0.154 nm) radiation at ambient conditions. Magnetic measurements were done by using superconducting quantum interference device (SQUID)-magnetometry (MPMS-XL-7, Quantum Design). The ac susceptibility measurements were performed by the same SQUID-magnetometer. Electrical resistivity was measured by using physical property measurement system (PPMS-9, Quantum Design).

## Results and discussion

**Figure 1:**
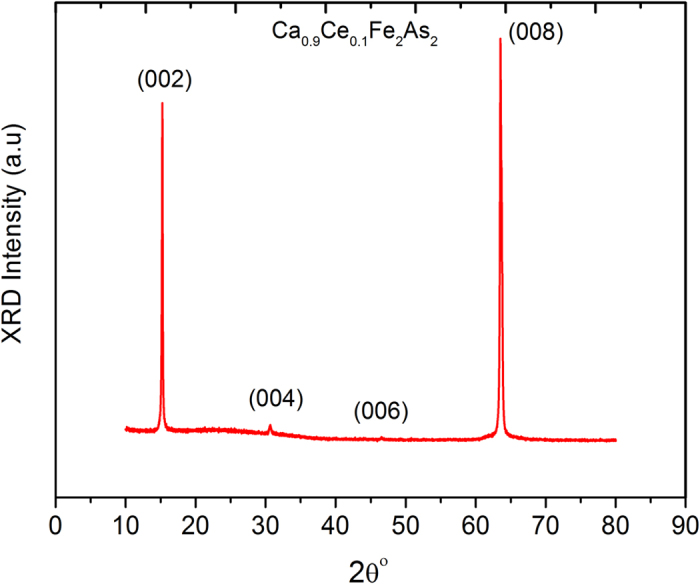
X-ray diffraction pattern of Ca_0.9_Ce_0.1_Fe_2_As_2_ single crystal.

Figure 1 shows the X-ray diffraction (XRD) pattern of Ca_0.9_Ce_0.1_Fe_2_As_2_ single crystal at ambient conditions. All the indexed peaks such as (002), (004, (006) and (008) are identified as the tetragonal ThCr_2_Si_2_ structure phase. There are no impurity phases detected in the XRD pattern. The calculated c-axis value for our sample is 11.6837A^o^ which is in accordance with the already reported value^24^.

**Figure 2:**
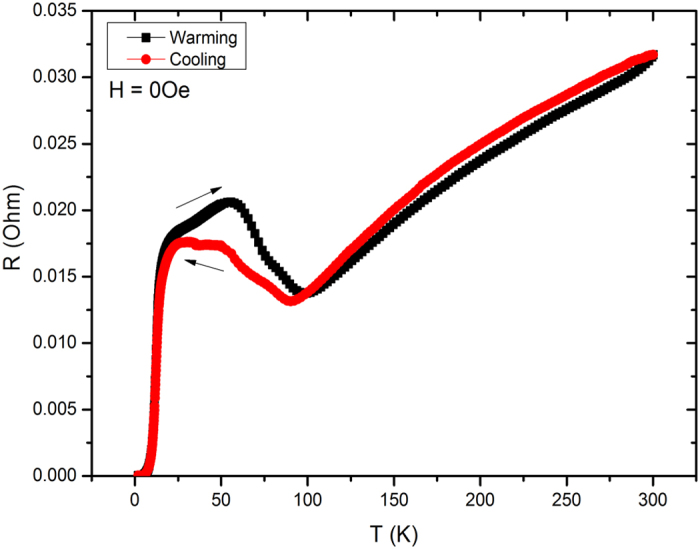
Cooling and warming temperature dependent resistance of Ca_0.9_Ce_0.1_Fe_2_As_2_ single crystal under zero applied field.

Figure 2 shows the electrical resistance of the Ca_0.9_Ce_0.1_Fe_2_As_2_ single crystal without any external field (H = 0 Oe) in cooling and warming temperature protocols as indicated by arrows. It clearly shows a superconductivity state at critical temperature (T_c0_) = 5 K. The rare-earth Ce ions doping at Ca sites induces superconductivity in CaFe_2_As_2_ parent compound. The lower value of T_c0_ shows the under-doping of Ce ions in CaFe_2_As_2_ compound, where superconductivity starts to emerge. A large R-T hysteresis of about 60 K in the cooling and warming data is associated with the first order tetragonal to orthorhombic structural transition along with simultaneous magnetic transition from paramagnetic to spin density wave (SDW) antiferromagnetic state of the Fe ions in the ab-plane^29^. The dip in the range 90 – 100 K is the onset temperature of SDW state. The R-T hysteresis can be also due to collapsed tetragonal (cT) phase as already reported in rare-earth doped CaFe_2_As_2_ superconductors^25^,^26^,^27^,^30^. However there is no cT phase observed up to 22% Ce doping in CaFe_2_As_2_ as reported by Saha *et al.*^24^. In our case, the c-axis also shows a small shrinkage due to only 10% Ce doping and hence the presence of cT phase can be ruled out. Therefore our under-doped Ca_0.9_Ce_0.1_Fe_2_As_2_ compound shows superconducting state along with a SDW transition.

**Figure 3:**
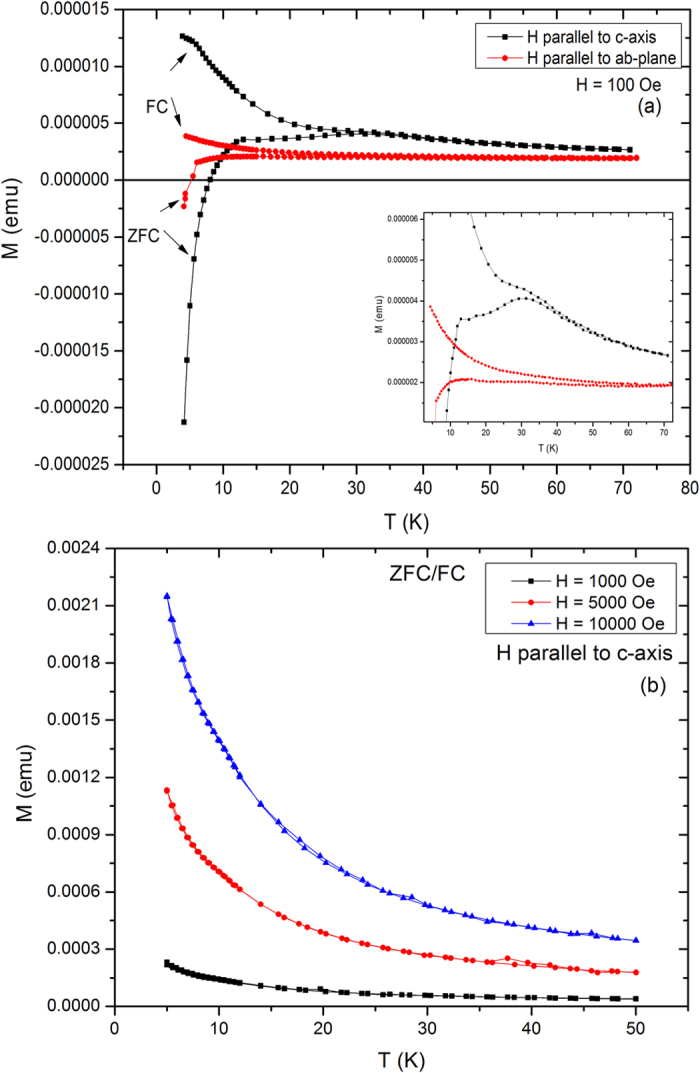
(**a**) ZFC/FC magnetization curves of the Ca_0.9_Ce_0.1_Fe_2_As_2_ single crystal at applied field (H) = 100 Oe parallel to c-axis and ab-plane, (**b**) ZFC/FC magnetization curves of the Ca_0.9_Ce_0.1_Fe_2_As_2_ single crystal at applied fields (H) = 1000 Oe, 5000 Oe and 10000 Oe parallel to c-axis.

Now we will move to magnetization studies to investigate the magnetic response of the system. Figure 3(a) shows the ZFC/FC magnetization of the Ca_0.9_Ce_0.1_Fe_2_As_2_ single crystal at applied field (H) = 100 Oe parallel to c-axis and ab-plane. For H // c-axis, the ZFC curve peaks at 32 K which is absent in case of H // ab-plane. The small ZFC peak is also evident in the *inset* of Fig. 3(a). The FC magnetization is higher for H // c-axis which is due alignment of the Ce spins along the c-axis. The magnetic anisotropy can be associated with the magnetic ordering/response of the Ce spins in the c-axis direction. Below 32 K for H // c-axis, the bifurcation of the ZFC/FC curves occurs with a sharp increase of FC curve and then FC plateau at low temperatures, which could be due to the presence of spin-glass like behavior^20^,^31^,^32^,^33^. Therefore ZFC peak may be associated with the onset of the spin-glass like behavior due to magnetic disorder and magnetic frustration of Ce spins in the Ca layer^14^,^31^,^34^. The frustration of the Ce spins can be due to their inhomogeneous distribution on the Ca lattice sites OR their interactions with the AFM aligned Fe spins in the ab-plane. As ZFC/FC splitting is present for both field directions, therefore ZFC/FC splitting can be also due to filamentary superconductivity. It is reported in the literature that CaFe_2_As_2_ parent compound exhibits filamentary superconductivity below 15 K^26^. Therefore we cannot rule out the existence of filamentary superconductivity (small area superconducting) in Ca_0.9_Ce_0.1_Fe_2_As_2_ with an increase of transition temperature up to 32 K with 10% Ce doping as compared to low temperature filamentary superconductivity in CaFe_2_As_2_ parent compound. The ZFC curve also shows a diamagnetic response below 6 K due to the superconducting state (Meissner effect) of the system which is in agreement with the R-T measurements (see Fig. 2). Figure 3(b) shows the ZFC/FC magnetization curves at higher applied fields (H) = 1000, 5000, and 10000 Oe parallel to c-axis. The ZFC/FC splitting, diamagnetic response and ZFC peak all vanish at higher applied magnetic fields. The weak Ce-Fe or Ce-Ce exchange interactions which are responsible for slow spin dynamics vanish at higher magnetic fields and Ce spins show paramagnetic behavior. Therefore, a slightly higher magnetic field destroys the weak spin-glass like state.

**Figure 4:**
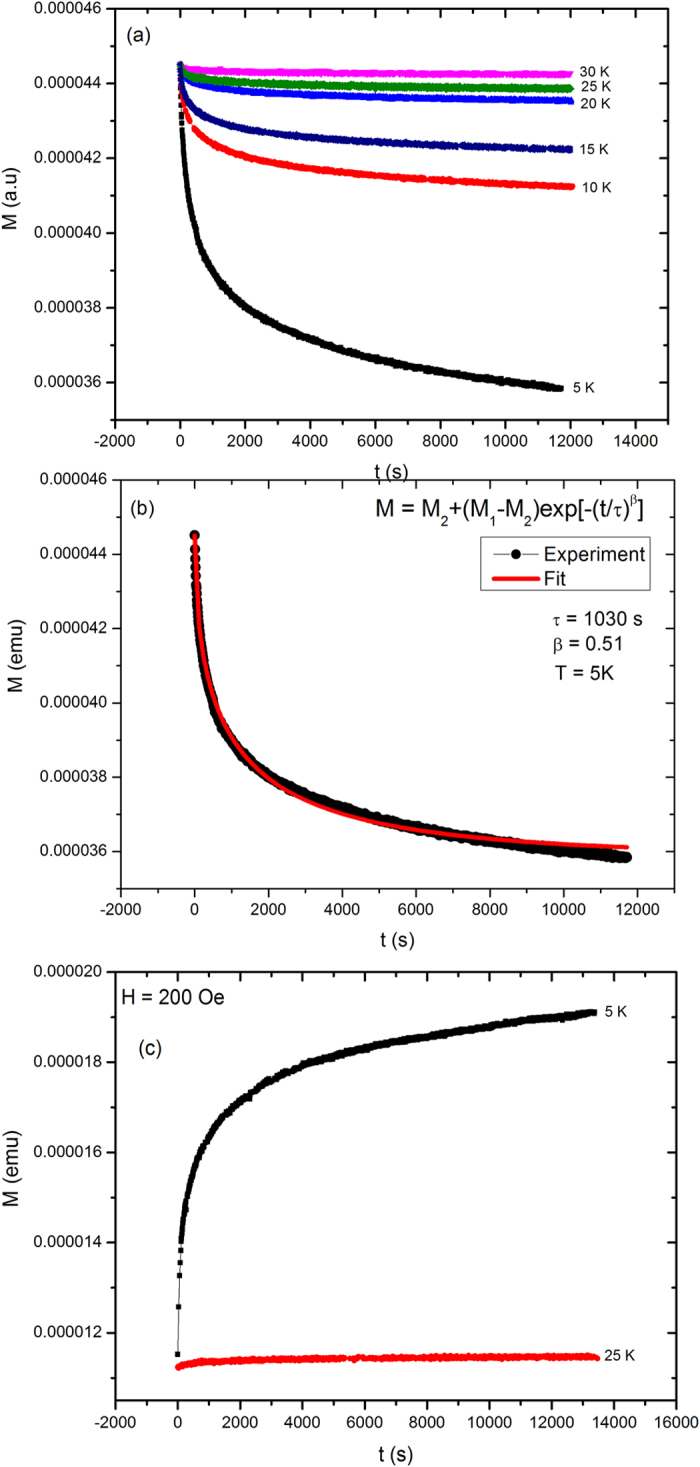
(**a**) FC (@ 200 Oe) relaxation curves of Ca_0.9_Ce_0.1_Fe_2_As_2_ single crystal at different temperatures (T) = 5, 10, 15, 20, 25, and 30 K, (**b**) stretched exponential law fit (solid line) to FC relaxation curve at 5 K, and (**c**) ZFC relaxation curves of Ca_0.9_Ce_0.1_Fe_2_As_2_ single crystal at temperatures (T) = 5 and 25 K.

To further investigate the magnetic response of the system, we have performed time dependent magnetic relaxation under FC and ZFC protocols at different temperatures. Figure 4(a) shows the time dependent FC (@ 200 Oe) relaxation curves of Ca_0.9_Ce_0.1_Fe_2_As_2_ single crystal at different temperatures (T) = 5, 10, 15, 20, 25, and 30 K. *The curves are vertically shifted for comparison*. For FC relaxation, the sample is FC in 200 Oe to target temperature (5, 10, 15, 20, 25, and 30 K). Afterwards the field is switched off and magnetization is recorded with time. It is interesting to note that at low temperatures, the magnetization does not relax after switching off the field and continuously decreasing, which indicates the presence of slow spin relaxation in the system^35^. In the literature there are two models usually used to fit the relaxation data for spin-glass or disordered systems; (i) logarithmic relaxation decay model and (ii) stretched exponential decay model^35^,^36^. We have fitted one of the relaxation curve recorded at 5 K using stretched exponential law as given below,
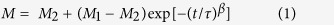
where M_1_ and M_2_ are initial and final magnetization, τ is the mean relaxation time and β is the shape parameter. Figure 4(b) shows the stretched exponential law fit (solid line) to the relaxation curve recorded at 5 K by using Eq. 1. The best fit parameters are τ = 1030 s and β = 0.51. The shape parameter (β) lies between 0 and 1 for different disordered systems. Spin-glass systems usually exhibit β in the range 0.2–0.6, below the freezing temperature^37^. Wang *et al.*^38^ reported the β value equal to 0.52 for the spin-glass alloy La-Fe-Mn-Si, below freezing temperature. The long relaxation time and value of β indicate the presence of slow spin relaxation in our system.

Superparamagnetic systems show slow spin relaxation in the FC protocol only, while spin-glass systems show slow dynamics in both ZFC and FC protocols^39^. Therefore we have also investigated magnetic relaxation in ZFC protocol at 5 K and 25 K as shown in Fig. 4(c). For ZFC relaxation, the sample was first ZFC to target temperature (in our case 5 K and 25 K) and then 200 Oe field is applied and magnetization is recorded with time. It is evident that the system also shows slow spin relaxation in ZFC protocol in addition to the FC protocol. Therefore our system shows slow spin relaxation in both FC and ZFC protocols which suggests the presence of spin-glass like behavior.

**Figure 5:**
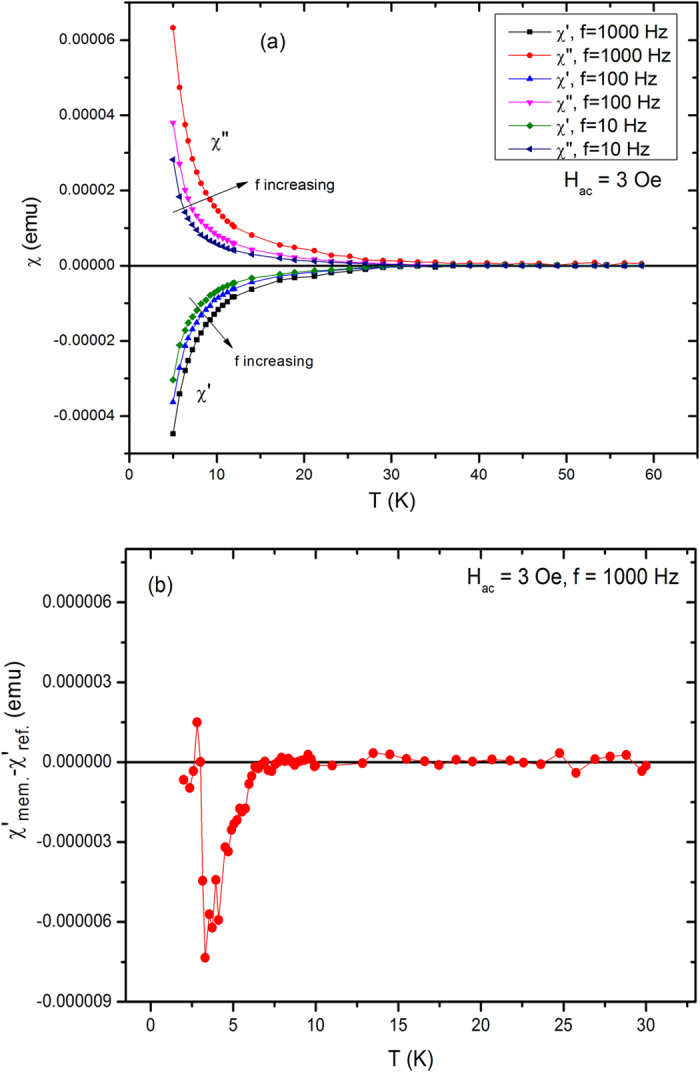
(**a**) Frequency dependence of in-phase and out-of-phase ac susceptibility of Ca_0.9_Ce_0.1_Fe_2_As_2_ single crystal, (**b**) memory dip due to difference of in-phase ac susceptibility scans. The sample is halted at 4 K for 2 h during the ZFC to get the memory curve, whereas the reference curve is determined without any halting temperature.

Spin-glass and superconducting state transitions usually show peak in the temperature dependence of in-phase/out-of-phase and out-of-phase ac susceptibility, respectively. Figure 5(a) shows the frequency dependence of ac susceptibility of Ca_0.9_Ce_0.1_Fe_2_As_2_ single crystal in the temperature range 5 – 60 K. In our case, ac susceptibility does not reveal any peak related to spin-glass OR superconducting state transition, which may be due to overlapped transition temperatures. The absence of spin-glass peak in the ac susceptibility can be also due to presence of strong diamagnetic background of the superconducting phase. There is frequency dependence in both in-phase and out-of-phase ac susceptibility but it does not provide any other useful information.

Spin-glass system usually show memory effect in ZFC protocol only, which is also considered as one of fingerprint for spin-glass behavior. To investigate the memory effect, one needs two curves, (i) the reference curve and (ii) the memory curve (for which the system is halted at particular temperature for a specified time). The difference between ZFC memory and ZFC reference curves exhibits a dip at the halting temperature due to presence of memory effect as recognised for spin-glass systems^40^. For the reference curve, the sample is continuously ZFC from 100 K to 2 K and then immediately the ac susceptibility (at 3 Oe alternating field and frequency f = 1000 Hz) is recorded on increasing temperature up to 30 K. For the memory curve, the sample is also ZFC to a certain waiting temperature (in our case 4 K) and halted there for some specific time (in our case 2 h). After this, the cooling is resumed to 2 K and the ac susceptibility is recorded on increasing temperature. Figure 5(b) shows the difference of the in-phase ac susceptibilities as a difference between memory and reference curves at ac field amplitude (H_ac_) = 3 Oe and frequency (f) = 1000 Hz in the temperature range 2 – 30 K. The system shows corresponding memory dip near the halted temperature which is the signature of memory effect^41^,^42^. The magnetization of memory curve decreases near the halted temperature because the system remembers of its originally disordered magnetic state near the halted temperature.

**Figure 6:**
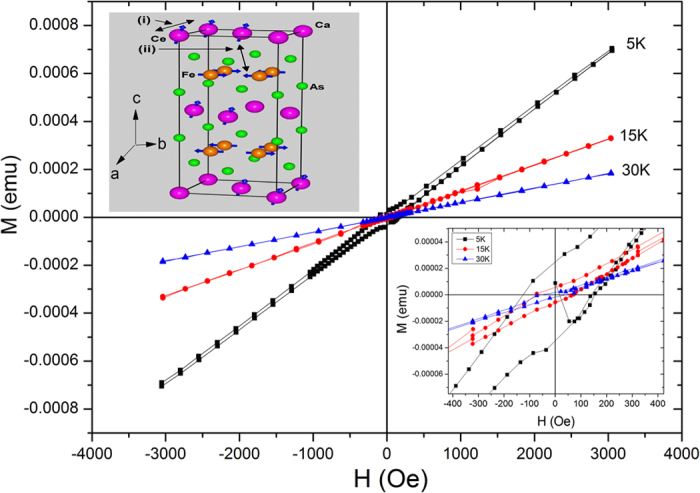
M-H loops of Ca_0.9_Ce_0.1_Fe_2_As_2_ single crystal at different temperatures (T) = 5 K, 15 K, and 30 K. Lower inset shows the coercivity region. Upper inset shows the demonstration of two possible origins of frustration of Ce spins responsible for slow spin dynamics; (i) random magnetic exchange interactions between the inhomogeneously distributed Ce spins ordered along the c-axis direction OR (ii) magnetic exchange coupling between Fe and Ce spins which leads to frustration among the Ce spins.

Figure 6 shows the M-H loops of Ca_0.9_Ce_0.1_Fe_2_As_2_ single crystal under ± 3000 Oe at different temperatures (T) = 5 K, 15 K, and 30 K. All the M-H loops are not saturated and magnetization increases with increasing applied field. The small hysteresis OR opening of M-H loops vanishes at higher magnetic fields which is in agreement with the high field ZFC/FC M-T curves (see Fig. 3(b)). Usually spin-glass systems show large coercivity such as for nanoparticles, while we get small coercivity for our system which is consistent with the literature for similar systems^13^. Ren *et al.*^13^ reported a small value of coercivity of about 20 Oe at 2 K for ferromagentically (T_c _= 20 K) aligned Eu spins in superconducting EuFe_2_(As_0.7_P_0.3_)_2_ single crystal. Marik *et al.*^43^ also reported a small value of coercivity of about 50 Oe at 4 K in the spin-glass state for cuprate Mo_0.3_Cu_0.7_Sr_2_TmCu_2_O_y_. In our case, coercivity (H_c_) increases from 70 Oe to 140 Oe as the temperature decreases from 30 K to 15 K. At 5 K, the diamagnetic response in the virgin curve (Meissner effect) confirms the superconducting state of the system as shown in the lower *inset* of Fig. 6, which is in agreement with T_c0_ in R-T and M-T measurements (see Figs. 2 and 3(a), respectively). The features such as appearance of H_c_ and open loop at 5 K are consistence with the bifurcation of ZFC/FC M-T curves and are typical for spin-glass systems. At T = 30 K, H_c_ and ZFC/FC splitting both nearly vanish due to unblocking of the disordered frozen magnetic Ce spins.

There can be two types of magnetic orders in Ca_0.9_Ce_0.1_Fe_2_As_2_, one is the AFM ordering of Fe spins in the ab-plane and other is the ordering of Ce spins along the c-axis (see inset of Fig. 6). The slow spin dynamics can be associated with one OR both of them. The two main ingredients of the slow spin dynamics OR spin-glass behavior are disorder and magnetic frustration. Considering the AFM ordering of Fe spins, the possibility of disorder and frustration among the Fe spins in the ab-plane can be ruled out because ZFC peak is observed only for case H // c-axis (see Fig. 3(a)). Therefore the anisotropic behavior of ZFC magnetization in Fig. 3 (a) suggests that the spin-glass like behavior originates from randomly distributed Ce spins in the Ca layer. It is reported that Ce spins exhibit magnetic order at low temperatures in different compounds^14^,^44^,^45^. Luo *et al.*^14^ reported FM and AFM ordering of Ce spins in CeFeAs_1−*x*_P_*x*_O_0.95_F_0.05_ (0 ≤ x ≤ 1) superconductor at low temperatures for different doping levels (x). Zhang *et al.*^45^ reported the Fe-Ce magnetic coupling and AFM ordering of Ce spins in CeFeAsO compound. In our case, the origin of the frustration from randomly distributed Ce spins can be due to two reasons, (i) complex magnetic interactions among randomly distributed Ce spins in the Ca layer which create frustration among Ce spins OR (ii) magnetic interactions between AFM aligned Fe spins (in the ab-plane) and random Ce spins (ordered in the c-axis) which can also create frustration among Ce spins (see upper inset of Fig. 6).

## Conclusions

In summary, coexistence of slow spin dynamics and superconductivity in Ca_0.9_Ce_0.1_Fe_2_As_2_ single crystal has been reported. The system also exhibits some features similar to spin-glass behavior but rather weak, therefore we call it *spin-glass like behavior*. The system shows a ZFC/FC splitting with an anisotropic (for H // c) ZFC peak at 32 K followed by a FC plateau at low temperatures. Magnetic relaxation in both the ZFC and FC protocols ensures the presence of slow spin dynamics in the system and suggests the presence of spin-glass like behavior. ZFC ac memory effect was also observed with a corresponding memory dip in the vicinity of the halted temperature. The origin of the slow spin dynamics can be due to frustration of the randomly distributed Ce spins which results either from the frustrated magnetic interactions between Ce spins OR the frustrated magnetic interactions between AFM ordered Fe and Ce spins.

## Additional Information

**How to cite this article**: Nadeem, K. *et al.* Slow Spin Dynamics in Superconducting Ca_0.9_Ce_0.1_Fe_2_As_2_. *Sci. Rep.* **5**, 10700; doi: 10.1038/srep10700 (2015).
